# Celsr3 Is Required for Normal Development of GABA Circuits in the Inner Retina

**DOI:** 10.1371/journal.pgen.1002239

**Published:** 2011-08-11

**Authors:** Alaron Lewis, Neil Wilson, George Stearns, Nicolas Johnson, Ralph Nelson, Susan E. Brockerhoff

**Affiliations:** 1 Department of Biochemistry, University of Washington, Seattle, Washington, United States of America; 2 Basic Neurosciences Program, National Institute of Neurological Disorders and Stroke, National Institutes of Health, Rockville, Maryland, United States of America; University of Pennsylvania School of Medicine, United States of America

## Abstract

The identity of the specific molecules required for the process of retinal circuitry formation is largely unknown. Here we report a newly identified zebrafish mutant in which the absence of the atypical cadherin, Celsr3, leads to a specific defect in the development of GABAergic signaling in the inner retina. This mutant lacks an optokinetic response (OKR), the ability to visually track rotating illuminated stripes, and develops a super-normal b-wave in the electroretinogram (ERG). We find that *celsr3* mRNA is abundant in the amacrine and ganglion cells of the retina, however its loss does not affect synaptic lamination within the inner plexiform layer (IPL) or amacrine cell number. We localize the ERG defect pharmacologically to a late-stage disruption in GABAergic modulation of ON-bipolar cell pathway and find that the DNQX-sensitive fast b1 component of the ERG is specifically affected in this mutant. Consistently, we find an increase in GABA receptors on mutant ON-bipolar terminals, providing a direct link between the observed physiological changes and alterations in GABA signaling components. Finally, using blastula transplantation, we show that the lack of an OKR is due, at least partially, to Celsr3-mediated defects within the brain. These findings support the previously postulated inner retina origin for the b1 component and reveal a new role for Celsr3 in the normal development of ON visual pathway circuitry in the inner retina.

## Author Summary

Visual information is transmitted through the retina from photoreceptors to bipolars to ganglion cells, the output neurons connecting to the brain. This vertical transmission of information is modulated by inhibitory lateral interneurons. Normal vision requires the proper transmission and processing of these neuronal signals. In the inner retina, amacrine cells are the main class of inhibitory interneurons. They modulate the information from bipolar to ganglion cells and are functionally responsible for adjusting image brightness and for detecting motion. Physiological studies have revealed important aspects of the mechanisms of inhibitory modulation, and anatomical studies have identified the many amacrine subclasses and their non-random arrangement within the retina. Although cell–cell interactions are thought to be critical for establishing the important physiological and morphological features of this cell class, the precise molecules and their functions are mostly unknown. In this paper we report the discovery of a mutant that identifies the atypical cell adhesion molecule, Celsr3, as critical for proper development of GABA-signaling pathways in the inner retina.

## Introduction

The vertebrate retina is a well-established model system for the study of neural circuit formation within the central nervous system. Synaptic circuits in the retina transform light information detected by photoreceptors into signals that retinal ganglion cells send to the brain. Processes of lateral interneurons within the retinal plexiform layers modulate the vertical transfer of information. Establishing precise synaptic connections is critical for the correct transfer of information. This process is highly specific and occurs in a precise temporal sequence after cells have established their proper retinal locations. The final maturation of synapses leads to normal adult circuits. One hypothesis is that self-avoidance is important in this process requiring members of the cadherin and immunoglobulin superfamilies [1]. However, the molecular identity of the critical family members and their mechanism in this process is largely unknown.

Celsr3 is an atypical 7-pass cadherin receptor. The ectodomain is comprised of multiple cadherin domains, EGF repeats and also laminin A G-type repeats. A seven transmembrane domain connects this with a G-protein binding intracellular signaling domain. Celsr3 is one of three vertebrate homologs of the *Drosophila* protein, Flamingo/Starry night, originally identified as critical in planar cell polarity [2], [3], dendritic outgrowth, branching and routing [4]. Recent papers analyzing *Celsr1–3* in mammalian nervous system development suggest that the functions of *Drosophila* Flamingo have been subdivided into the 3 *Celsr* genes. By early postnatal stages these genes define distinct regions of the developing nervous system [5], [6]. CELSR3 plays multiple critical roles in brain development; it suppresses neurite growth in hippocampal neurons [7], is critical in axonal tract formation in the CNS [8]–[11] and is essential for proper interneuron migration in the mouse forebrain [12]. The precise molecular mechanisms underlying these functions are not yet known. The role of Celsr3 in the vertebrate retina has not been investigated.

In this report we describe a new zebrafish mutant with a defect in correct signal processing within the retina due to a premature stop codon within the *celsr3* gene. *celsr3* mutants lack an OKR, the ability to track rotating illuminated stripes and develop a super-normal b-wave in the ERG. In wild type (WT) animals, *celsr3* is abundant in amacrine and ganglion cells in the retina. We find that a developmentally late inhibitory modulation of ON-bipolar cell transmission is disrupted when this protein in missing. Loss of Celsr3 does not cause gross changes in retinal cell morphology or lamination. Quantification of GABA receptor number reveals an increase over normal in the mutant on ON-bipolar cells. Finally, we demonstrate that additional abnormalities with the mutant brain contribute to the lack of an OKR. We conclude that Celsr3 is important in maturation of inhibitory circuits within the inner retina, and our findings reveal a new role for this protein.

## Results

### *zvm7^w65^* disrupts cone visual circuitry at late stages of retinal development

At one week of age, zebrafish larvae rely on cone photoreceptors for the initiation of vision. Thus, behavioral assays that measure visual responses of larvae target cone visual pathways. One behavioral assay that has been used extensively to identify visually impaired zebrafish is the OKR [13]–[15]. This assay measures the ability of zebrafish larvae to track rotating illuminated stripes [16]–[19]. Many mutants identified with this assay have defects within the retina and some have specific defects in cones [20]–[22].

**Figure 1:**
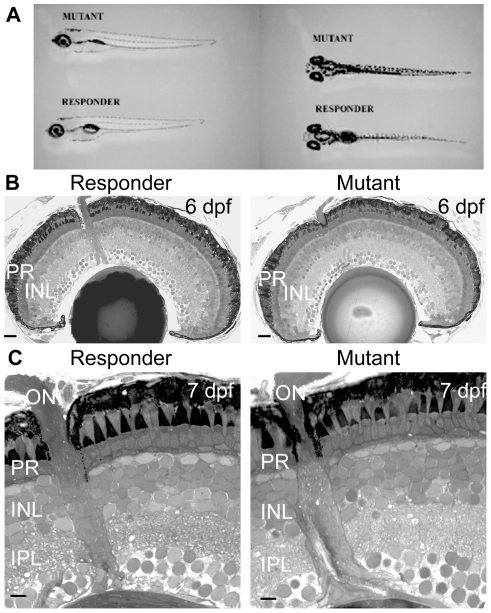
The morphology and retinal histology of the *zvm7^w65^* mutant appears normal. A) Mutant fish at 5 dpf do not develop a swim bladder but appear otherwise normal. B and C) The histology of the eye appears normal in the *zvm7^w65^* mutant. B) 6 dpf retinas. Scale bars are 20 µm C) magnification of 7 dpf retinas. Scale bars are 5 µm. PR, photoreceptors; INL, Inner Nuclear Layer; IPL, inner plexiform layer.

Here we describe a new mutant, *zvm7^w65^* isolated by OKR behavioral screening (see Materials and Methods). At 5 days postfertilization (dpf), one fourth of larvae generated from heterozygous parents did not have an OKR response, indicating a recessive mutation. In addition, these larvae did not develop a swim bladder, a general marker of fish health, and died by 10 dpf. Otherwise, at 5 and 6 dpf the mutant larvae appeared normal in morphology; overall body length and both brain and eye shape and size were normal (Figure 1A). Further, spontaneous eye movements occurred at the same frequency in WT and mutants indicating that the loss of OKR is not due to a defect in muscular control or function (data not shown).

**Figure 2:**
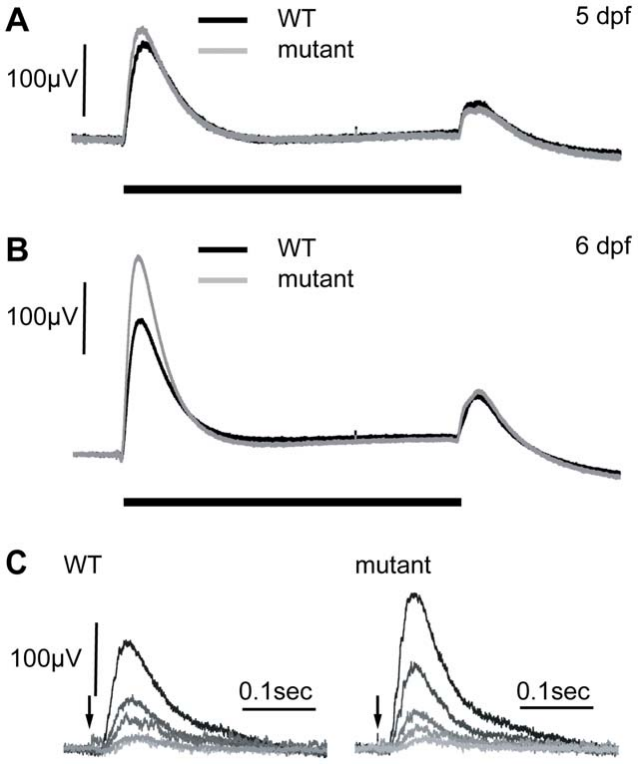
Electroretinograms show an increase in the b-wave response in mutants at 6 dpf. A) At 5 dpf, WT and *zvm7^w65^* responses are similar. B) At 6 dpf, *zvm7^w65^* eyes develop an increase in the b-wave. The b-wave was 176±13 µV in the WT (n = 30) and 260±48 µV in the *zvm7^w65^* fish at 6 dpf (n = 32) (p<.001). A) and B) show an average representative trace including at least 9 animals. C) WT and *zvm7^w65^* eyes have a similar light threshold response. Eyes were exposed to a millisecond light flash (arrow) at intensities differing by 0.5 log units (brightest = .41 mW). Amplitudes of mutant b-waves were larger at all light levels. Images show traces from a single representative animal.

To determine if *zvm7^w65^* affects the retina, we analyzed retinal histology and recorded ERGs from WT and *zvm7^w65^* mutant larvae. The retina appeared normal in histological sections. All of the layers of the retina were present and there were no signs of cellular degeneration or death (Figure 1B and 1C). Thus, our mutation does not cause gross changes in retinal morphology. Further, at 5 dpf a normal ERG response was recorded (Figure 2A). The ERG response is divided into several characteristic features corresponding to various aspects of the visual response. These are the small negative a-wave that occurs immediately after lights on and is associated with the photoreceptor response. The a-wave is followed by a large positive b-wave response, which consists primarily of the ON-bipolar response modulated by amacrine and horizontal cell inputs [23], [24]. At lights off there is an additional wave, the d-wave, which originates from the OFF-bipolar response. Using a prolonged flash (3 sec.) we found that both the ON and OFF components of the ERG were normal in *zvm7^w65^* fish at 5 dpf (Figure 2A).

We went on to measure the ERG of WT and *zvm7^w65^* fish at 6 dpf. Remarkably we discovered that in *zvm7^w65^* mutant fish the b-wave increases dramatically over the WT response at this age (Figure 2B). The increase occurs specifically in the amplitude of the ON component of b-wave and does not affect either the latency of the ON response or any aspect of the d-wave OFF response. In these semi-saturating records (approximately 2 log units above threshold), the peak amplitude of the b-wave was 176 µV±13 std in the WT (n = 30) and 260 µV±48 std in the *zvm7^w65^* fish at 6 dpf (n = 32) (p<.001). To determine if increased photoreceptor sensitivity was the cause of the increase in the ERG response we compared the light-sensitivity of WT and mutant larvae. No difference in visual threshold was found between mutant and WT (Figure 2C). The normal ERG threshold response suggests that the source of the enhanced b-wave lies post-synaptic to photoreceptors possibly within the metabotropic responses of ON-bipolar cells, or neural elements directly post-synaptic to these cells.

### *zvm7^w65^* has a mutation in the zebrafish *celsr3* gene

**Figure 3:**
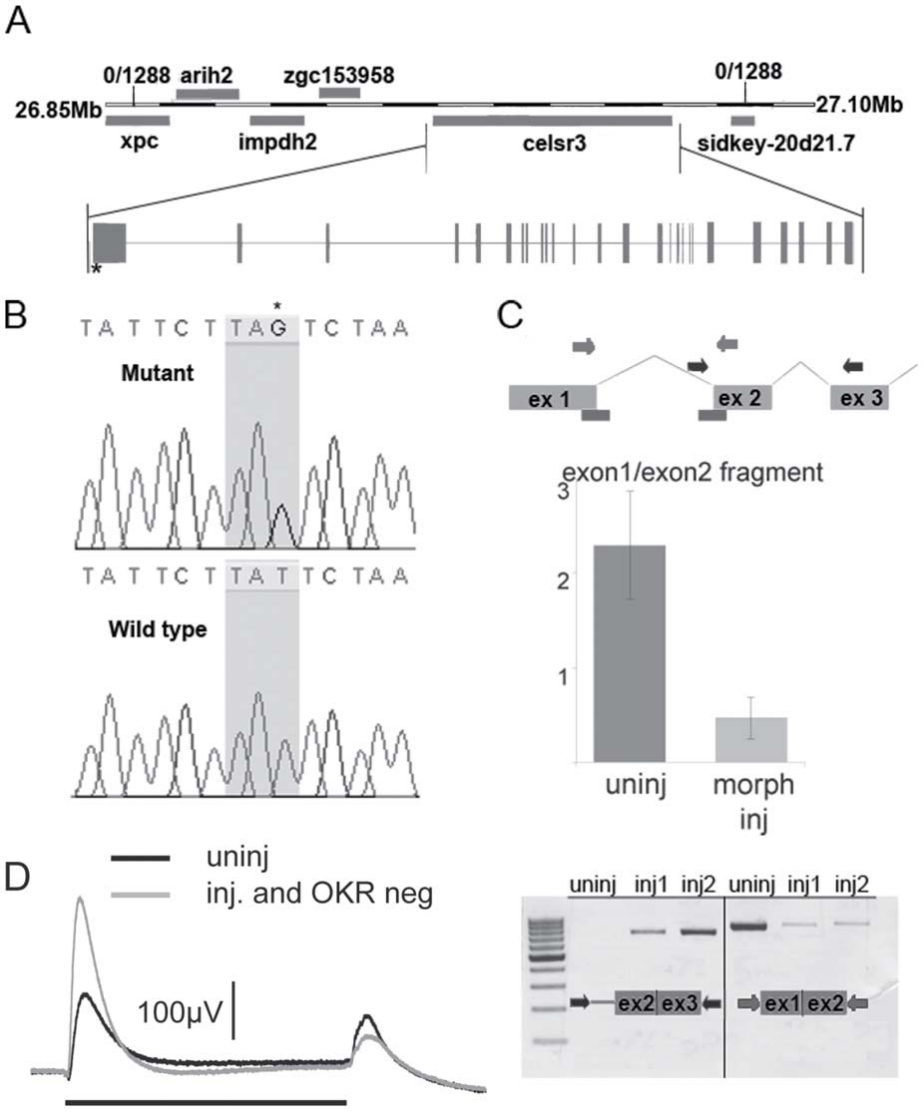
*zvm7^w65^* has a mutation in *celsr3* gene. A) A diagram of the genetic locus of *celsr3*. B) The *zvm7^w65^* fish have a mutation in *celsr3* that creates a stop codon at nt postion 651 of exon1. C) A diagram of predicted morpholino interaction sites (bars). Injection of splice site morpholinos results in abnormal splicing of *celsr3* mRNA. Levels of correctly spliced *celsr3* were determined using primers to exon1 and exon2 in a qPCR rxn (middle panel) and by agarose gel electrophoresis (bottom panel right side). Incorrect splicing can be seen in morpholino injected (inj) animals using primers in intron 1 and exon3, which do not give a product in the uninjected (uninj) animals (left bottom panel). D) Morpholino injected *zvm7^w65^* heterozygotes that are OKR-negative have an increased b-wave (n = 4) compared to uninjected siblings. Black bar indicates 3 sec. light pulse.

Using bulk segregant analysis we mapped *zvm7^w65^* to chromosome 8. We then used a mutant panel of 1288 larvae and localized the mutation between genome markers G47365 and G39328 (http://uswest.ensembl.org/Danio_rerio/Info/Index). We refined our mapping panel data by identifying single nucleotide polymorphisms and then analyzing these within our recombinants. To identify the *zvm7^w65^* mutation we isolated cDNA from mutant and WT larvae and sequenced several genes in the region defined by zero recombinants. Using this method we identified a significant single nucleotide change only in one gene, *celsr3* (Accession:XM_001922677.3). This mutation introduces a premature stop codon very early in the gene at nucleotide position 651 (Figure 3A and 3B). To determine if the mutant message was subject to nonsense-mediated decay we did quantitative RT-PCR analyses. The level of *celsr3* mRNA remained the same in mutant larvae compared to WT (data not shown).

*celsr3* is a large gene containing 39 exons encoding a 3646 amino acid protein. It is a member of the cadherin superfamily. Celsr3 stands for cadherin EGF LAG seven-pass G-type receptor 3. The zebrafish version shares 52.2% amino acid sequence identity with mouse CELSR3. However, it contains a unique amino-terminus. The premature stop codon in *zvm7^w65^* is within the unique N-terminus (Figure 3A and 3B).

We used morpholino knockdown to confirm that the loss of Celsr3 was the source of the OKR and ERG phenotypes. Morpholinos transiently block message splicing or translation and are commonly used to produce complete loss-of-function or hypomorphic phenotypes early in development. Injection of 2 different splice-site morpholinos caused abnormal processing of the *celsr3* mRNA (Figure 3C). As larval zebrafish age the morpholino effect is diluted and is often significantly decreased by 4–6 dpf when the OKR and ERG tests are performed. To potentially sensitize larvae to reductions in Celsr3 we injected splice site morpholinos into eggs from crosses between adult fish heterozygous for *zvm7^w65^* and WT animals, resulting in mixed WT and heterozygous clutches. We reasoned that the heterozygous fish would already have a decreased level of Celsr3 protein and might therefore be more sensitive to additional reductions due to the morpholino. Normally larvae heterozygous for *zvm7^w65^* are both OKR positive and show normal WT-like ERG recordings. When splice site morpholinos that target the exon1 – intron1 boundary of *celsr3* were injected into these mixed eggs, 38% of the fish were OKR negative. We determined the genotypes of eight of these OKR negative fish and found, as predicted, that all eight were heterozygous for the *celsr3* mutation. In contrast, 6 of 8 fish that were OKR positive after morpholino injection were WT. Further, morpholino injected fish that were OKR negative had a higher b-wave than either morpholino injected siblings that were OKR positive or uninjected siblings (Figure 3D and data not shown). Thus, injection of splice-site morpholinos reduced the amount of normally spliced *celsr3* message and reproduced in genotypically heterozygous fish both the OKR and ERG phenotype detected in the homozygous mutant. Given the uniqueness of the ERG phenotype (no other reported mutant has this phenotype) and the severity of the identified mutation (a premature stop codon), we conclude that *celsr3* is the gene mutated in *zvm7^w65^* fish.

### *celsr3* mRNA is abundant in the inner retina and ganglion cell layer

**Figure 4:**
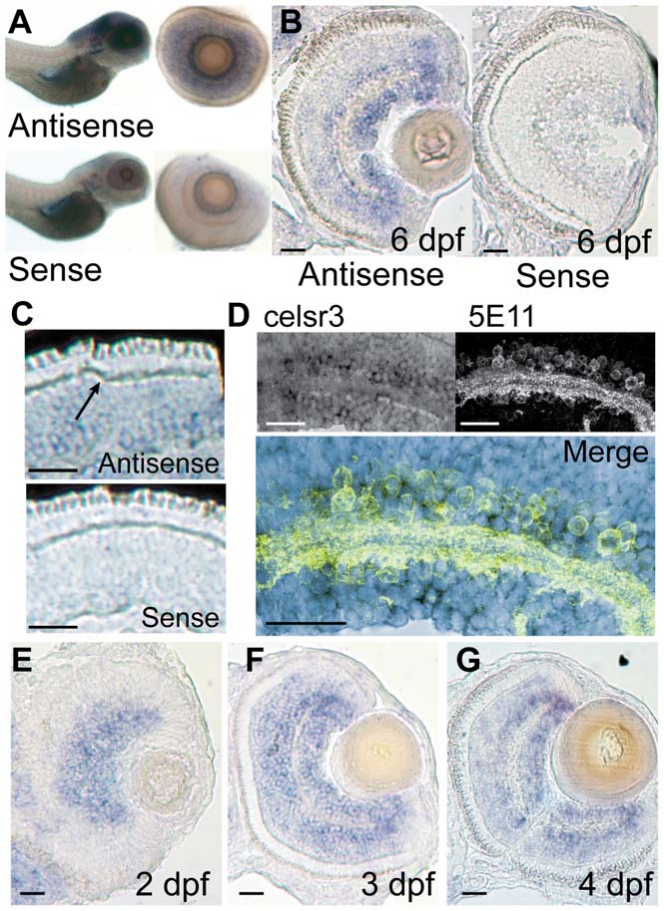
*celsr3* is abundant within amacrine and ganglion cell layers of the retina. In situ hybridization was performed on WT animals using probes for exon1 of *celsr3*. A) Whole mounts of 6 dpf fish show localization to the brain and eye. When eyes were removed they show localization in the INL and ganglion cell layer. B and C) Cryosections of a 6 dpf eye show staining in the INL with abundance in the amacrine and ganglion cell layers. Sense controls show no staining. Arrow in C points to unlabeled horizontal cells. D) Slides were probed for *celsr3* message and then probed with the anti-amacrine antibody 5E11 confirming presence of the message within amacrine cells. E–G) A time series of *celsr3* message localization shows accumulation around the IPL at all ages from 2–4 dpf. Scale bars are 20 µm.

To determine which cells were likely responsible for the enhanced ERG b-wave in *celsr3* mutant fish, we conducted *in situ* hybridization (ISH) experiments and localized the *celsr3* mRNA. We used two different RNA probes for these experiments: a probe within the first exon and a probe in the 3′UTR region of the transcript. Both probes gave similar results (data not shown). In whole mount *in situ* hybridization both of these probes showed staining in the brain and eye at 5 dpf (Figure 4A). There was little to no expression in the tail or body regions. Within the eye staining appeared primarily in the inner nuclear layer (INL) and ganglion cell layers (Figure 4A and 4B).

To more accurately identify the layers in the eye that expressed *celsr3* we cryosectioned 6 dpf animals and performed the ISH on retinal sections. WT animals were grown in 1-phenyl-2-thiourea to prevent the development of pigment that might obscure staining in the eye. Fish were fixed and frozen and cut into 16 µM sections and then probed for *celsr3* mRNA (see Materials and Methods). *celsr3* transcripts were present at low levels throughout the INL and appeared more abundant in two layers above and below the IPL (Figure 4B). These two layers are consistent with the cellular localization of amacrine and ganglion cells within the retina. In contrast, *celsr3* message appeared completely absent from the photoreceptor layer and from horizontal cells (Figure 4C). The lack of staining in photoreceptors and horizontal cells suggests Celsr3 is not functioning within these cells.

The predominant *celsr3* staining at 6 dpf is in amacrine and ganglion cells (Figure 4B). Within the amacrine and ganglion cell layers, the *celsr3* staining was most prominent in the periphery of the eye where many cells are strongly stained. In the central areas of the eye a majority of cells in the ganglion cell layer are stained (Figure 4B). Some cells at the lower edge of the IPL did not stain strongly for *celsr3*, and these may be displaced amacrine cells (Figure 4B). In the amacrine cell layer there is also a variety of staining between cells, suggesting that some amacrines may express *celsr3* while others do not. To further confirm that the cells expressing *celsr3* within the INL were amacrine cells we combined the slide ISH procedure with immunohistochemistry for amacrine cells using the 5E11 antibody. This antibody recognizes an unknown antigen found in most amacrine cells within the fish [25]. The combination of these two protocols showed that *celsr3* expressing cells were indeed the amacrine cells in the INL (Figure 4D). Further, staining below the IPL extends beyond the displaced amacrine cells, confirming that ganglion cells also express *celsr3* (Figure 4D).

Since the ERG phenotype develops by 6 dpf we were also interested to see if there were changes in the localization of *celsr3* over time. Slices were prepared from 2, 3, and 4 dpf animals and probed for *celsr3* localization. At 2 dpf the IPL is just beginning to form. The *celsr3* staining was robust around the forming IPL, and in the layers that were becoming amacrine and ganglion cells (Figure 4E). At 3 dpf the staining spread around the IPL and a light but clear staining of some bipolar cells was also apparent (Figure 4F). The staining patterns for 4 dpf animals was similar (Figure 4G). This staining throughout the central and inner INL and in the ganglion cell layer persisted in retinal sections from 6 dpf animals (Figure 4D). These data suggest that Celsr3 functions in amacrine, bipolar and ganglion cells in the retina. Further, although the ERG phenotype develops over time, the expression of *celsr3* was similar at all ages examined. Loss of WT maternal RNA could also not explain the phenotype since previous work on this gene demonstrated a lack of expression in the early embryo [26]. One might have anticipated that the onset or change in expression of *celsr3* could have coincided with the development of an aberrant b-wave in the mutant. This was not the case. Thus, the development of the ERG phenotype is probably not due to an alteration in the presence of Celsr3, but possibly to the alteration of some normal developmental programming or modification within the retina that depends on this protein.

### The loss of Celsr3 does not broadly affect the organization of the IPL

In other organisms the loss of Celsr3 has a variety of effects including defects in interneuron migration, dendritic pathfinding, axonal tract formation and several others [2]–[4], [8], [9], [11], [12]. Many of these defects lead to morphological changes that are evident with careful cellular analysis. Since the effect of *celsr3* mutations on the retina has not been previously analyzed, we used antibodies to characterize many different cell types within the retina. We focused primarily on amacrine cells since *celsr3* message was abundant in these cells and changes in these cells could explain the enhanced b-wave phenotype detected in *zvm7^w65^* mutants. We also examined ON-bipolar cells and Müller glia. Cell bodies were counted in sections that showed a portion of the optic nerve ensuring that the same regions of the eye were counted. The counts are shown as cells per 10 µM thick eye section (see Materials and Methods).

**Figure 5:**
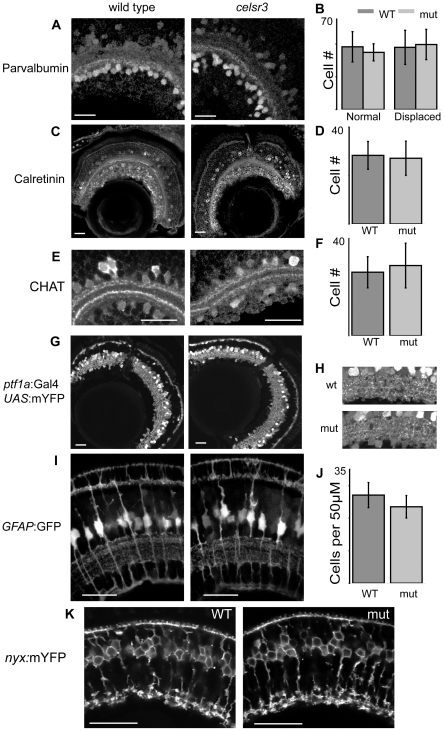
Cell localization and IPL organization are unchanged in the *celsr3* mutant. A) α-parvalbumin labels all displaced amacrine cells and a subpopulation of amacrines in the INL. B) counts of parvalbumin cells, displaced and normal amacrines were counted separately C) α-calretinin labels a subpopulation of amacrines in the INL and all ganglion cells. D) Counts of calretinin positive amacrine cells. E) α-CHAT stains a subpopulation of amacrine cells that laminate in 2 major and 2 minor sublaminae within the IPL. All sublaminae are present in the mutant. F) counts of CHAT positive amacrine cells in the INL G) Tg(*ptf1a:*Gal4VP16, *UAS:*mYFP) animals express mYFP in all amacrine and horizontal cells. H) Close ups of the IPL in Tg(*ptf1a:*Gal4VP16, *UAS:*mYFP) animals I) Images of Tg(*GFAP:GFP*) animals, which express GFP in all Müller cells. J) Counts of Müller cells per 50 µM. K) Tg(*nyx:*mYFP) animals express mYFP in the ON-bipolar cells. Scale bars are 20 µm.

Three antibodies were used to identify amacrine subtypes. The parvalbumin antibody labels a major band within the IPL as well as all displaced amacrines and some amacrines in the INL (Figure 5A). The two populations were counted separately and their numbers did not change between WT and mutant animals (Figure 5B). Further, the strong band of parvalbumin staining in the ON layer of the IPL is present in both mutant and WT sections (Figure 5A). The small calcium binding protein calretinin is found in a small population of amacrine cells in the INL and in all of the ganglion cells of the zebrafish [27]. It also labels a major band within the IPL, which displayed no change in mutant versus WT animals (Figure 5C). The subset of amacrine cells in the INL labeled by the calretinin antibody was counted and these numbers did not change (Figure 5D). Finally, the CHAT antibody was used to examine a small population of amacrine cells and several sublaminae in the IPL. The CHAT antibody marks four sublaminae in the IPL: two in the OFF and two in the ON layers. All four of these laminae can be seen in both the mutant and WT animals (Figure 5E). Further, the number of CHAT positive amacrine cells in the INL did not change in the mutant as compared to WT (Figure 5F).

To examine the entire amacrine and horizontal cell populations simultaneously we crossed the *zvm7^w65^* mutation into the Tg(*ptf1a:*Gal4VP16, *UAS:*mYFP) lines. This line labels all horizontal and amacrine cells [28], [29]. Mutants showed no obvious differences from WT in these lines (Figure 5G). In these animals the density of cell bodies prevented the accurate counting of cells, but this fluorescent expression does allow visualization of several broad lamina within the IPL (see magnified image, Figure 5H). These laminae were present in both mutant and WT animals and no obvious morphological changes were noted (Figure 5H).

Several years ago regulation of extracellular potassium by Müller glia had been hypothesized to contribute to the b-wave of the ERG response [30]. However, more recent work using mutant animals that lack the inwardly rectifying potassium channel (Kir4.1) showed that the b-wave in these animals is unchanged [31]. To confirm that Müller cells were not visibly affected in our mutant we crossed *zvm7^w65^* fish with the transgenic line Tg(GFAP:GFP) expressing GFP in Müller cells [32]. Müller cells appeared normal with laminations around the photoreceptors, in the IPL and at their end feet (Figure 5I). Further, we counted the density of cells using 3D reconstructions in intact live retina and found that the density was unchanged compared to WT (Figure 5J).

The other major group of cells that could cause an increase in the b-wave are the ON-bipolar cells. To visualize the majority of ON-bipolar cells in our animals we crossed them into transgenic fish expressing Tg(*nyx:mYFP*) [33]. The Tg(*nyx:mYFP*) animals express mYFP in most of their ON-bipolar cells. This line shows no major differences between WT and mutant. Specifically, the ON-bipolar cell boutons reside in three sublaminae toward the bottom of the IPL (Figure 5K). The morphology of these cells appeared normal. Thus, no major changes were identified in cells likely to cause a change in the b-wave.

### Celsr3 is needed for proper inhibitory modulation in the inner retina

**Figure 6:**
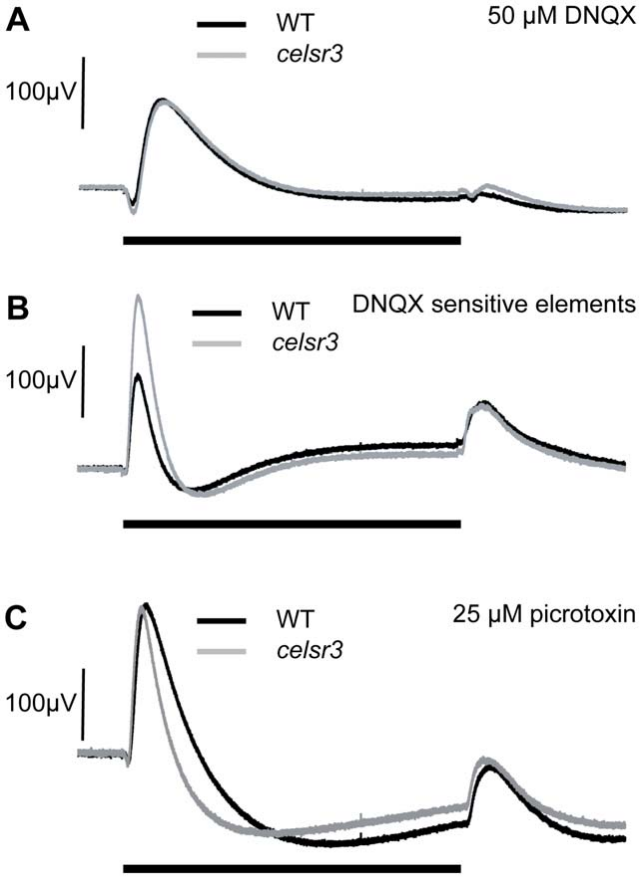
ERGs at 6 dpf in the presence of drugs suggest alterations in GABAergic signaling. A) In 50 µM DNQX, which isolates the ON–bipolar cells, WT and *celsr3* mutant eyes have a similar response suggesting that ON-bipolar cells are normal (see Results), and it is ionotropic glutamate responses that are defective. B) The DNQX sensitive curve is obtained by subtracting the DNQX-treated waveform from the untreated response [23]. The isolated DNQX sensitive b-wave element is called b1. The b1 element is larger in *celsr3* mutants. C) The WT and *celsr3* mutant response is similar in 25 µM picrotoxin, a GABA_A/C_ inhibitor, suggesting that changes to GABA signaling are causing the increase in the *celsr3* mutant b1-wave. Black bar indicates 3 sec. light pulse. Graphs are the average of at least 6 animals.

Celsr3 is abundant within the INL of the retina. However, loss of Celsr3 protein does not alter the number of cells within various amacrine subpopulations, and both amacrine cells and ON-bipolar cells continue to develop normal sublaminae within the IPL. As an alternative strategy to determine the role of Celsr3 in modulating cone signaling, we treated mutant and WT eyes at 6 dpf with several inhibitors to isolate aspects of the ERG response. Since the photoreceptors were functioning normally and the primary changes were to the b-wave, we started with the AMPA/kainate receptor antagonist 6,7-dinitroquinoxaline-2,3-dione (DNQX), which blocks both the interaction of the OFF-bipolar cells with the photoreceptors and the majority of the input to amacrine, horizontal and ganglion cells. Thus, in DNQX, remaining transmission is from photoreceptors to ON-bipolar cells. Using DNQX, we found that the ON-bipolar b-wave responses of the mutant were very similar to WT. The peak amplitude of the b-wave in DNQX is 133 µV±35 std in WT (n = 12) and 136 µV±70 std in mutant (n = 13) (p = 0.9; Figure 6A). This finding indicates that the enlargement of the metabotropic b-wave response detected in mutants is generated through ionotropic glutamate pathways. In other words, the abnormality in the mutant b-wave results from cell types whose responses are blocked by DNQX, namely amacrine, ganglion or horizontal cells. Importantly, since *celsr3* localizes to the inner retina and is not found in horizontal cells, these pathways would appear to be inner retinal pathways, some of which could be amacrine cells that modulate ON-bipolar cell axon terminals [34]. The ON-bipolar metabotropic synapse does not appear to be affected by the mutation.

To identify the different components of the b-wave we subtracted the ERG in the presence of DNQX from the ERG in the absence of DNQX. The remaining waveform represents the DNQX sensitive portion of the ERG, an initial component of the b-wave called the b1-wave [23]. This wave has been hypothesized to result from ON-bipolar synaptic currents with amacrine and/or ganglion cells within the inner retina [23]. Our mutant is specifically causing an increase in this b1 component of the ERG (Figure 6B). This is the first mutation known to specifically increase this ERG component and represents a powerful tool for investigating the source of the b1 wave within the retina.

To determine whether inhibitory circuits were being altered, we used the GABA_A/C_ antagonist picrotoxin to remove GABAergic input to the visual signal. In zebrafish, picrotoxin affects many aspects of the signaling between the amacrine and bipolar cells including local feedback inhibition and longer-range lateral inhibition. In the presence of picrotoxin, the b-wave in WT is increased and this increase is followed by a large slow hyperpolarization. The d-wave is also slightly increased (Figure 6C).

In the presence of picrotoxin, the mutant and WT ERGs were also not distinguishable. The peak b-wave in picrotoxin was 208 µV±85 std for WT (n = 10) and 201 µV±123 std for mutants (n = 11) (p = 0.9; Figure 6C). In the mutants, this means the b-wave did not increase in the presence of the drug and may, in fact, have decreased slightly. The similarity of WT and mutant ERGs in the presence of picrotoxin, together with the localization of *celsr3* in the inner retina, suggests the changes within the eye involve alterations in the GABAergic connections between the bipolar and amacrine cell populations.

### GABA receptor number is increased in the mutant ON-bipolar terminals

**Figure 7:**
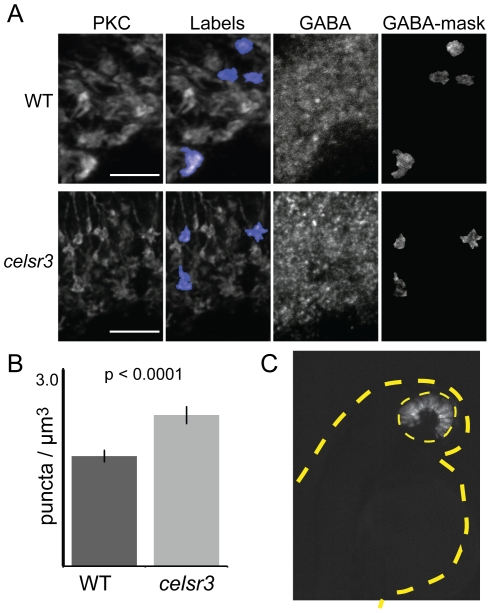
The number of GABA receptor puncta is increased on *celsr3* mutant ON-bipolar terminals. A) ON-bipolar cells in cryosection were stained with anti-PKC antibodies and anti-GABAγ2. The PKC label fields were overlayed on the GABA signal, and the number of puncta was counted in each terminal rotated and visualized in 3D. Image shows merged z-stacks. Scale bars are 5 µm. B) The GABA puncta in the ON-bipolar terminals per µm^3^ for WT and *celsr3* mutants (n = 60 terminals for each). Error bars are standard error. C) Mosaic 2 dpf larva containing WT cells in the eye (yellow dashed line) of a *celsr3* mutant animal. Despite a large WT eye field transplant, this animal remained OKR negative. The tan dashed line outlines larva body.

We hypothesized that an alteration in sensitivity to GABA could underlie the physiological changes detected by ERG. One way this could occur would be directly, through chloride (Cl^−^) currents flowing through GABA receptors on bipolar-cell axon terminals. Such currents may constitute an inner retina contribution to the ERG b-wave. To test this directly, we quantified receptor number on ON-bipolar terminals. For these experiments we used an antibody directed against the gamma 2 subunit of GABA receptors [35]. Binding and functional studies suggest that the γ subunit is a component of both A and C receptors on teleost bipolar cells [36], [37]. In order to count the number of GABA receptors on the ON-bipolar axon terminals we co-labeled cells for PKC. Cyrosections were labeled for PKC and GABAγ2, imaged, and then images were renamed and randomized to ensure blind sampling. We then created 3D label fields of individual bipolar terminals, and used this as a mask to count the number of GABA receptor aggregates that were present on each terminal (Figure 7A). Finally, images were identified as either mutant or non-mutant. Using this method we found that the average number of GABA receptor puncta/µm^3^ was 2.02±0.11 ste in the WT and 2.77±0.15 ste in the mutant (n = 60 for each; P<0.0001; Figure 7B). On average this results in one additional GABA puncta per terminal. This finding indicates that there are direct changes in components of GABA signaling within the mutant that may be responsible for the physiological changes detected by ERG.

### *celsr3* mutants also have defects within the brain

Finally, we asked why *celsr3* mutants lack an OKR. The OKR measures the ability of zebrafish larvae to track rotating stripes. This activity requires both motion-sensitive circuits of the eye, created by the amacrine and ganglion cells, and areas of the brain involved in processing this information. It is possible that *celsr3* mutants lack an OKR because motion-sensitive circuits within the retina are malfunctioning. However, it is also possible that the lack of an OKR is due, at least partially, to abnormalities within the brain, where *celsr3* is also abundantly expressed (Figure 4A).

To determine whether the lack of an OKR was due exclusively to defects within the eye, we conducted blastula transplantation experiments and evaluated whether a mostly WT eye was sufficient to rescue vision in an otherwise mutant fish (i.e. mutant brain). We generated mosaic fish with large WT transplants within the eye. We then analyzed the OKR and genotyped these fish (see Materials and Methods). As a proof of principle for this experiment, we rescued the cone degeneration mutant *pde6c* [21] with large clones of WT cells transplanted into an otherwise mutant fish (n = 2). In contrast, none of the 12 *celsr3* mutants we examined showed any restoration of the OKR response. Of these mutant animals, 4 had WT transplants that covered at least 50% of the eye. A representative *celsr3* mutant with a large WT transplant exclusively in the eye is shown in Figure 7C. Although this experiment does not rule out a retinal contribution to the lack of an OKR, it does indicate that additional defects within the brain of *celsr3* mutants likely play a role in the OKR defect. This can explain why animals at 5 dpf, with a normal ERG, are OKR negative.

## Discussion

In this paper we present the first characterization of the role of Celsr3 in the vertebrate retina. We exploit a newly identified zebrafish mutant lacking Celsr3. The important findings from this study are that 1) *celsr3* mRNA localizes to the amacrine and ganglion cell layers of the retina, 2) Celsr3 is required for normal GABAergic modulation of the ON-bipolar response 3) loss of Celsr3 does not lead to gross changes in retinal cell number or cellular lamination 4) GABA-receptor number on ON-bipolar terminals is increased in *celsr3* mutants and 5) *celsr3* mutants likely have additional circuitry defects within the brain. Little is known about the molecular cues required for inhibitory circuit formation in the retina and our study indicates that Celsr3 plays an important role in this process.

Signaling between cells within the developing nervous system is required on multiple levels including: differentiation of appropriate numbers of neural types from progenitors, proper migration of cells to the appropriate location, initiation and growth of axonal and dendritic projections, identification of appropriate synaptic partners and refinement and maturation of synaptic contacts [38]. One family of cell signaling molecules that has been identified as important for these processes is the *celsr* family [38]. Similar to cadherins, these genes contain extra-cellular cadherin repeats, which are important for cell-cell interactions. Unlike standard cadherins, they also contain a seven-pass transmembrane receptor. In vertebrates, there are three members of the *celsr* family, and they each have primary functions in different aspects of cell polarity and cellular interaction. Specifically, mutations in mouse *Celsr3* are lethal and mice die at birth due to central hypoventilation [8]. In addition, the loss of CELSR3 results in a variety of extensive changes throughout the brain including a loss of major axon tracts and changes in interneuron number and migration [12]. Because these animals die before eye opening, no studies have been made analyzing the effects of *celsr3* mutations on the eye.

Celsr3 is a cell adhesion molecule and thus abnormal cellular interactions likely underlie the mutant phenotype. The two primary documented effects of *Celsr3* in the mouse brain are the loss of major axon tracts and a defect in interneuron migration [8], [12]. These two effects are not apparent in the eyes of *celsr3* mutant zebrafish. Histology of the eye showed that the optic nerve is still present in the *zvm7^w65^* mutant, and that the retina was laminated with no apparent cell death. We combined ISH for *celsr3* with an antibody stain for amacrine cells and found that a majority of amacrine cells expressed *celsr3* message. There are at least 28 different types of amacrine cells in the zebrafish eye [39]. Using antibody staining we have examined the cell numbers and positions of a variety of amacrine subpopulations and found no change in either of these parameters. Thus, the *celsr3* mutation does not have the same major effects on cell populations in the zebrafish eye as it does in the mouse brain.

We identified the *celsr3* mutant initially because it lacked an optokinetic response, the ability to track moving objects. This behavior has been extensively characterized in mammals and the neural pathways involved are known [40]. The initial detection is initiated in the retina and then processing occurs in several regions of the brain. Elegant behavioral and physiological studies in zebrafish have also localized the OKR to ON visual pathways [41] as well as subsets of neurons within the brain [42] and the current hypothesis is that the neural pathways underlying this reflex are similar in zebrafish and mammals [42]. We analyzed whether the lack of an OKR could be explained solely by defects in the retina or whether additional defects in the brain were also present in *celsr3* mutants. Although we were able to rescue vision of a photoreceptor degeneration mutant by placing large clones of WT cells within the mutant eye, we were unable to rescue the OKR in *celsr3* mutants with large transplants of WT cells. This finding indicates that zebrafish *celsr3* mutants have processing defects within the brain that are sufficient to eliminate an OKR behavioral response. Our finding does not rule out that the mutant retina may also contribute to the lack of an OKR. The necessity for Celsr3 in normal brain function is consistent with both the abundance of *celsr3* in the brain ([5], [10], [43] and Figure 4A) and the demonstrated critical role for this gene in the mouse brain [7]–[12]. This finding also establishes the zebrafish mutant reported here as useful for studying development of normal brain circuitry.

We established that *celsr3* mutants have a defect in retinal signal processing by ERG analysis. Similar to the mouse, loss of Celsr3 function in zebrafish is lethal and mutant animals die at 10 dpf. However, the visual system develops rapidly in zebrafish and larvae already have excellent visual function by 4 dpf. Thus, in zebrafish, the effect of a *celsr3* deficiency on retinal function can be evaluated. Using the ERG assay we found that, at 6dpf, eyes from *celsr3* mutants showed an increase in the major ON component of the ERG response, the b-wave. An increase in the b-wave is unique; to our knowledge no other mutation has this phenotype. As such, the *celsr3* mutant presents an important opportunity to dissect the molecular mechanisms underlying the formation of circuitry responsible for this component of the ERG. We used the AMPA/Kainate inhibitor DNQX to isolate the ON-bipolar cells and examine their light response in the absence of modification by circuitry within the retina [23]. In the presence of DNQX, the *celsr3* mutant response was identical to the WT response, suggesting that ON-bipolar function, in the absence of horizontal and amacrine modulation, is normal. We then used the GABA_A/C_ inhibitor picrotoxin to decrease GABAergic signaling. Using this drug, the WT and mutant responses were also similar. In zebrafish retina, picrotoxin blocks the GABAergic responses in many areas of the retina including ON-bipolar cells, both in their dendrites, and in their axon terminals [34]. In the inner retina, GABAergic amacrine cells modulate picrotoxin-sensitive GABA receptors on bipolar cell axon terminals [44]. Since we did not detect *celsr3* mRNA in horizontal cells, but found that it is abundant in amacrine cells, these results indicate that *celsr3* mutants have changes in the GABAergic connections between the ON-bipolar cells and amacrine cells.

**Figure 8:**
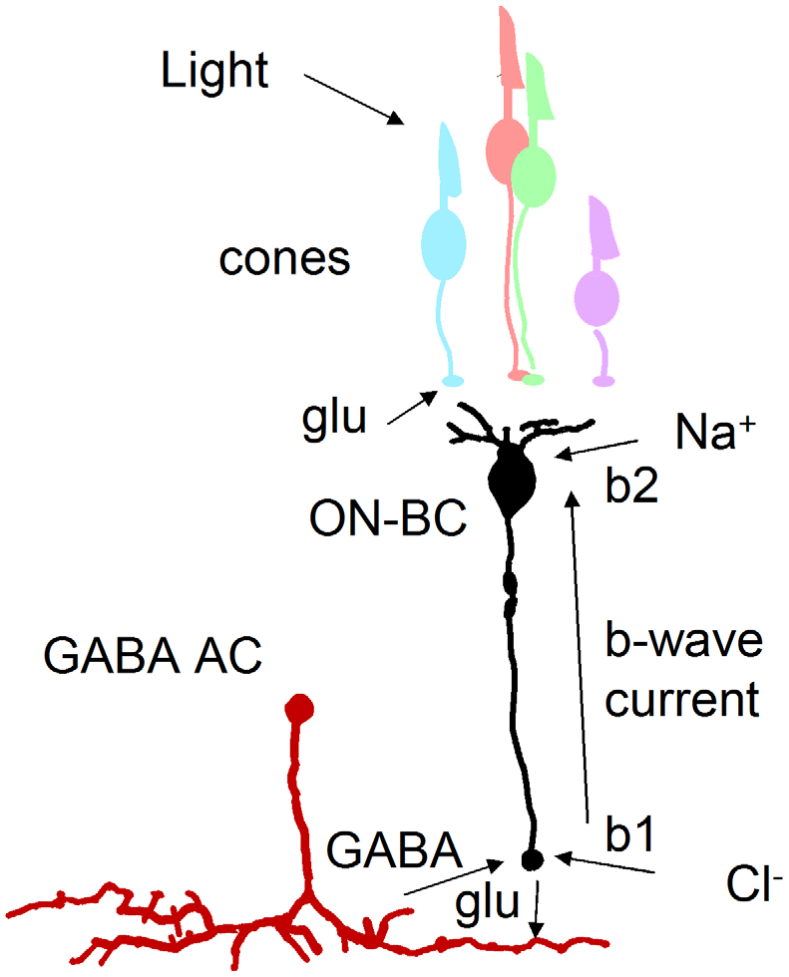
GABA-dependent mechanism for enlarged b-wave. Light leads to a decrease in glutamate (glu) in the cone synaptic cleft. This causes depolarization and release of glutamate from ON-bipolar cells (ON-BC) and consequent GABA release from amacrine cells (AC). The b-wave is a circulation of current around the ON-bipolar cell that is generated, in part, from Na^+^ ions flowing into dendrites and chloride ions (Cl^−^) flowing through GABA receptors into terminals. An increase in GABA receptor number in *celsr3* mutants would increase this current loop, resulting in an enlarged b-wave response.

An important finding explaining the physiological defects was our discovery that mutant ON-bipolar terminals have an increase in GABA receptors. The inhibitory effects of amacrine cells can be loosely divided into two categories: local feedback inhibition and longer-range lateral inhibition [45]. During feedback inhibition an amacrine cell that is stimulated by a particular bipolar terminal will then release GABA onto that terminal to cause inhibition. This is an extremely rapid response [34], [46] perhaps similar to the DNQX-sensitive b1 element of the b-wave (Figure 6B). This b1 element appears supernormal in the *celsr3* mutant at 6 dpf. A molecular explanation for this phenotype is the increase in GABA receptors. This increase would make a larger b-wave because positive charge flowing into ON-bipolar dendrites at light onset would result in the same sign of radial current flow as anion charges flowing into ON-bipolar terminals at light onset (see Figure 8). The block of b1 by DNQX is due to hyperpolarizaton of amacrine cells presynaptic to the bipolar terminal. One of the unusual aspects of our mutant is that picrotoxin addition does not increase the height of the later b-wave or b2 peak. This may correspond to a failure of slower lateral inhibition during the later peak response time of ON-bipolar cells. This delayed lateral modulation may be significantly abrogated in *celsr3* mutant animals. The increase in GABA receptor number may be indicative of larger changes in GABAergic signaling throughout the retina, possibly due to pathfinding or maturation defects.

Invertebrate studies have revealed additional subtle defects due to loss of atypical cadherins. Celsr3 is a vertebrate homolog of a *Drosophila* protein Flamingo. In the fly eye Flamingo concentrates between cells and influences axon trajectories and target selection through homotypic interactions [47], [48]. In the epidermis, loss of Flamingo function causes subtle defects in dendritic tiling, a process where homotypic interactions also play a role [49]. Given the necessity for even coverage within the visual field and the importance of retaining spatial information from the eye to the brain, it has long been assumed that most types of cells in the vertebrate eye tile [50]. While a variety of reporter genes for select ganglion cell types have recently been uncovered [51], the actual cell surface molecules responsible for tiling and stratification in the retina are less well known.

Within the zebrafish eye the cell bodies of horizontal cells are known to tile, and recent evidence has shown that the Müller glia form tiling territories in both the inner and outer plexiform layers [28]. However, the identification of different sub-types of amacrine and ganglion cells in zebrafish has not yet progressed to the point where extensive tiling has been characterized. Thus it was not possible to evaluate dendritic tiling in the current study. Although our work counting the density of amacrine and Müller glia did not show significant changes in the cell body locations of these cells (Figure 5), future investigations may uncover tiling defects in axonal or dendritic projections. These subtle morphological defects may cause the changes in receptor number and subsequent alterations in signaling detected in this study.

In conclusion, we have demonstrated that Celsr3 is critical for normal development of inhibitory circuits within the inner retina. We find that GABA modulation of ON-bipolars is enhanced due to a proliferation of GABA receptors on ON-bipolar terminals. Gross changes in cell number, position or morphology were not detected in mutant fish retina, and thus these types of changes are not implicated in causing this phenotype. Future studies will be directed toward analyzing subtle changes in dendritic and axonal tiling and well as changes in adhesiveness between Celsr3 containing cells.

## Materials and Methods

### Zebrafish maintenance and mutant isolation

Adult fish and larvae were maintained at 28.5°C in reverse-osmosis distilled water reconstituted for fish compatibility by addition of salts and vitamins [52] on a 10/14 h dark/light cycle. This study was carried out in strict accordance with the recommendations in the Guide for the Care and Use of Laboratory Animals of the National Institutes of Health. The protocol was approved by IACUC of the University of Washington.

The *zvm7^w65^* mutant was isolated in a three-generation screen of ethyl nitrosourea-mutagenized AB* strain zebrafish using the OKR behavioral assay as described previously [15], [17], [21], [53]. Progeny (between 4–6 dpf) from crosses between F2 siblings were partially immobilized in 6% methylcellulose (Sigma, St. Louis, MO), and their eye movements were analyzed in response to rotating illuminated stripes. In crosses between *zvm7^w65^* heterozygotes, 25% of the larvae showed no eye movements in white light. Fish did not track stripes under any light intensities or stripe widths examined (data not shown). There were no obvious phenotypic differences in electrophysiology or histology between WT and heterozygous *zvm7^w65^* fish. For experiments identifying and scoring polymorphisms, a hybrid strain between AB* and WIK was used (also see Results).

### Transgenic lines

To visualize ON-bipolar cells, fish heterozygous for the *zvm7^w65^* allele were mated to fish carrying the nyx:MYFP transgene. This transgene directs expression of the membrane-targeted form of YFP (yellow fluorescent protein) in a majority of ON-bipolar cells [33]. Amacrine cells were visualized with the *ptf1a:Gal4VP16* line previously described [28], [29], kindly provided by the Wong lab (UW, Biological Structure), crossed to the *UAS:*mYFP line also described [28]. The transgenic line *Tg*(*gfap*:GFP), kindly provided by Dr. Raymond (Univ of Michigan), expresses GFP in, all Müller Glia within the retina [32].

### Morpholino injections

Eggs from crosses between *zvm7^w65^* heterozygotes and WT animals were injected at the one cell stage with either splice-site morpholino 1.1 (EX1/INT1 5′-CTCCCGTTACTGAACTTACCAGTGA-3′) at 15 ng/µl or with a mixture of morpholino 1.1 at 10 ng/µl and morpholino 1.2 (INT1/EX2 5′-GCCATCTGAAAAACACACAGGACCA-3′) at 5 ng/µl. Injected eggs were raised to 5 dpf and then tested for blindness by OKR. The ERG response of animals separated into OKR positive and negative pools was then examined one day later at 6 dpf. These experiments were repeated three times.

### ERG recordings

Electroretinograms were recorded as described previously [24]. Briefly, 5 and 6 dpf larvae were anesthetized in tricaine and eyes were removed using a fine tungsten wire loop. Excised eyes were then placed in an oxygenated Ringer's solution (in mM; 130 NaCl, 2.5 KCl, 20 NaHCO_3_, 0.7 CaCl_2_, 1.0 MgCl_2_, and 20 glucose), and a glass electrode was positioned directly onto the cornea. After 3 minutes (min) of dark adaptation, eyes were exposed to white light flashes and their electrical responses recorded. Data was acquired and processed as described previously [54]. Peak values are listed as the mean ± standard deviation. All recordings are an average of at least 6 animals.

For drug treatments, fish at 6 dpf were allowed to swim in embryo media with the drug for 1 min, and then treated as above with Ringer's solution also containing the drug. Drugs were dissolved and stored as recommended by the manufacturer (Tocris bioscience, Ellisville, Missouri). Picrotoxin was used at 25 µM and DNQX at 50 µM. In most cases eyes showed the drug effect after the standard 3 min dark adaptation. However, for picrotoxin eyes required 8 min of dark adaptation before the drug effects stabilized. An 8 min dark adaptation did not change the ERG in the absence of drugs.

### Cryosections and light microscopy

Fish were grown to the indicated age in days, euthanized by immersion in ice, and then fixed in 4% paraformaldehyde (1× PBS, 3% sucrose) for 2 hrs at room temperature (rt) or overnight (o/n) at 4°C. Fixed fish were washed 1× in PBS and then immersed in 30% sucrose (1× PBS) o/n at 4°C. Fish were incubated in 50% OCT, 15% sucrose for 30 min at rt then frozen in 100% OCT on dry ice. 16–20 µM slices were cut using a Leica CM1850 cryostat.

Mutant and sibling OKR-positive larvae (a mixture of WT and heterozygotes) were observed by light microscopy as described previously [55].

### Immunohistochemistry (IHC) and cell counts

Slides were washed 3× with PBS, incubated at rt for 1 hr in blocking buffer (1×PBS, 0.3% Triton X-100, 3% lamb serum), and then treated with primary antibody in blocking buffer o/n at 4°C. Primary antibodies used were calretinin (Millipore, 1∶500), parvalbumin (Millipore, 1∶200), 5E11 (a generous gift from J. Fadool, 1∶50 [25]), and CHAT (Millipore, 1∶30). For the CHAT antibody slides were boiled for 10 min in 10 mM sodium citrate after the first PBS wash, and lamb serum was replaced with donkey serum. After primary incubation slides were washed 3× in PBS and then incubated with secondary antibody diluted (1∶1000) in PBS for 1 hr at rt. Secondary antibodies used were Alexiflour 568 and 488, anti-rabbit, goat and mouse (Invitrogen). Slides were washed 3× with PBS, mounted in Vectashield (Vector Laboratories), and imaged using a 60× objective (N.A. 1.35) and an Olympus FV1000 scanning confocal microscope.

Cell bodies were counted in sections that showed a portion of the optic nerve ensuring that the same regions of the eye were counted. Z-stacks were taken with a step size of 1 µM and the cells from 10 consecutive stacks were counted for each eye. The counts are shown as cells per 10 µM thick eye section. At least 6 eyes were counted for each condition. For Müller cell counts images were taken of live animals as described previously [28]. Images were rendered in 3D using the programs Metamorph (Medical Devices) and Amira (Visage Imaging), and cells were counted over a 50 µM area of retina.

### GABA receptor counts

Fish were fixed for 15 min in 4% paraformaldehyde and prepared for cyrosectioning. Cryosections of WT and mutant fish were immunolabled with 1∶300 rabbit anti-PKC β1 (Sigma sc-209, secondary anti-rabbit Alexiflour 488 1∶1000) and 1∶2500 anti-GABAγ2 (a generous gift from Prof. Jean-Marc Fritschy, University of Zurich; secondary anti-guinea pig Alexiflour 568). Slides were imaged with the Olympus FV1000, and the two colors were collected sequentially in 0.3 µm z stacks. Stacks were assembled in Amira and the 3D volume of PKC-labeled bipolar terminals was labeled. Only terminals that were completely encompassed by the image, and could be accurately separated from their neighbors, were labeled. 15–25 terminals were labeled per image. The 3D label field of the bipolar terminals was then used to mask the GABA signal. Dots were counted within the individual label fields in randomized blind samples. After counts were made, samples were categorized as either mutant or non-mutant. The volume of each terminal label field was also determined. The final value is an average of counts/µm^3^ ± standard error for 3 different fish and 60 total terminals from each genotype.

### In situ hybridization

In situs were performed using sense and antisense RNA probes to either Exon 1 (nt 2327–nt 3541) or the 3′UTR (434 bp fragment with 214 bp within the final exon) of *celsr3*. Whole mount and slide in situs were conducted as described previously [56], [57], except that for slide in situs the proteinase K step was not performed and 10% polyvinyl alcohol (MW 35,000–50,000; Sigma) was added to the NBT, BCIP staining solution. For IHC, ISH sections were postfixed for 20 min at rt in 4% paraformaldehyde, 1× PBS, before starting the IHC protocol.

### Blastula transplants

Blastula transplants were performed as previously described [58]. Images of the transplant were taken at 2 dpf using a Zeiss Discovery V12 epifluorescence stereomicroscope. The fish were allowed to grow to 5 dpf and were then tested for OKR and genotyped by sequencing.
